# Supplementary material for: Two common disease-associated TYK2 variants impact exon splicing and TYK2 dosage
Source: PLoS One. 2020 Jan 21;15(1):e0225289. doi: 10.1371/journal.pone.0225289 (PMC6974145; doi:10.1371/journal.pone.0225289)
Supplement: S1 Table — (PDF) [file pone.0225289.s001.pdf]

S1 Table
Summary of single-tissue eQTL for rs2304256 and rs12720270 on *TYK2* transcript expression across 48 tissues (GTEx release V8)

Tissue	rs2304256		rs12720270	
	NES	P-Value	NES	P-Value
Whole blood	0.11	6.9E-15	0.085	5.5E-07
Adrenal gland	0.42	2.9E-11	0.36	3.8E-06
Nerve-tibial	0.14	1.0E-10	0.14	8.2E-09
Skin-sun exposed (lower leg)	0.13	4.8E-10	0.12	2.1E-06
Artery-tibial	0.092	8.8E-07	0.089	4.3E-05
Skin-not sun exposed (suprapublic)	0.11	1.4E-06	NS	
Adipose (subcutaneous)	0.11	2.8E-06	NS	
colon (sigmoid)	0.15	6.7E-06	0.15	6.4E-05
Lung	0.096	1.8E-05	NS	
Breast-mammary tissue	0.14	2.2E-05	0.15	4.0E-05
Brain-cerebellum	0.21	2.8E-05	NS	
Esophagus-gastroesophageal junction	NS		0.16	2.6E-05
Other tissues	NS		NS	

NES: normalized effect size
NS: not significant
https://gtexportal.org/home/snp/rs2304256 **https://gtexportal.org/home/snp/rs12720270**
