# Supplementary material for: Two common disease-associated TYK2 variants impact exon splicing and TYK2 dosage
Source: PLoS One. 2020 Jan 21;15(1):e0225289. doi: 10.1371/journal.pone.0225289 (PMC6974145; doi:10.1371/journal.pone.0225289)
Supplement: S1 Fig — Partial exon 7 and exon 9 sequences are boxed. The central exon 8 encodes the 66 aa segment. Red arrowhead points to Val362. (PDF) [file pone.0225289.s002.pdf]

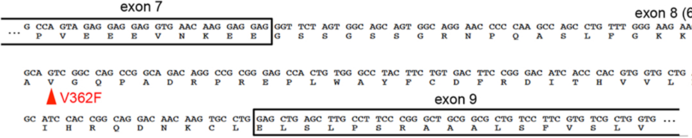

exon 7
exon 8 (66aa)
... G CCA GTA GAG GAG GAG GTG AAC AAG GAG GAG GGT TCT AGT GGC AGC AGT GGC AGG AAC CCC CAA GCC AGC CTG TTT GGG AAG AAG GCC AAG GCT CAC AAG
P V E E E V N K E E G S S G S S G R N P Q A S L F G K K A K A H K
GCA GTC GGC CAG CCG GCA GAC AGG CCG CGG GAG CCA CTG TGG GCC TAC TTC TGT GAC TTC CGG GAC ATC ACC CAC GTG GTG CTG AAA GAG CAC TGT GTC A
A V G Q P A D R P R E P L W A Y F C D F R D I T H V V L K E H C V S
V362F
exon 9
GC ATC CAC CGG CAG GAC AAC AAG TGC CTG GAG CTG AGC TTG CCT TCC CGG GCT GCG GCG CTG TCC TTC GTG TCG CTG GTG ...
I H R Q D N K C L E L S L P S R A A A L S F V S L V

S1 Fig
