# Supplementary material for: Two common disease-associated TYK2 variants impact exon splicing and TYK2 dosage
Source: PLoS One. 2020 Jan 21;15(1):e0225289. doi: 10.1371/journal.pone.0225289 (PMC6974145; doi:10.1371/journal.pone.0225289)
Supplement: S1 Raw images — (PDF) [file pone.0225289.s004.pdf]

Fig. 2A (raw images)

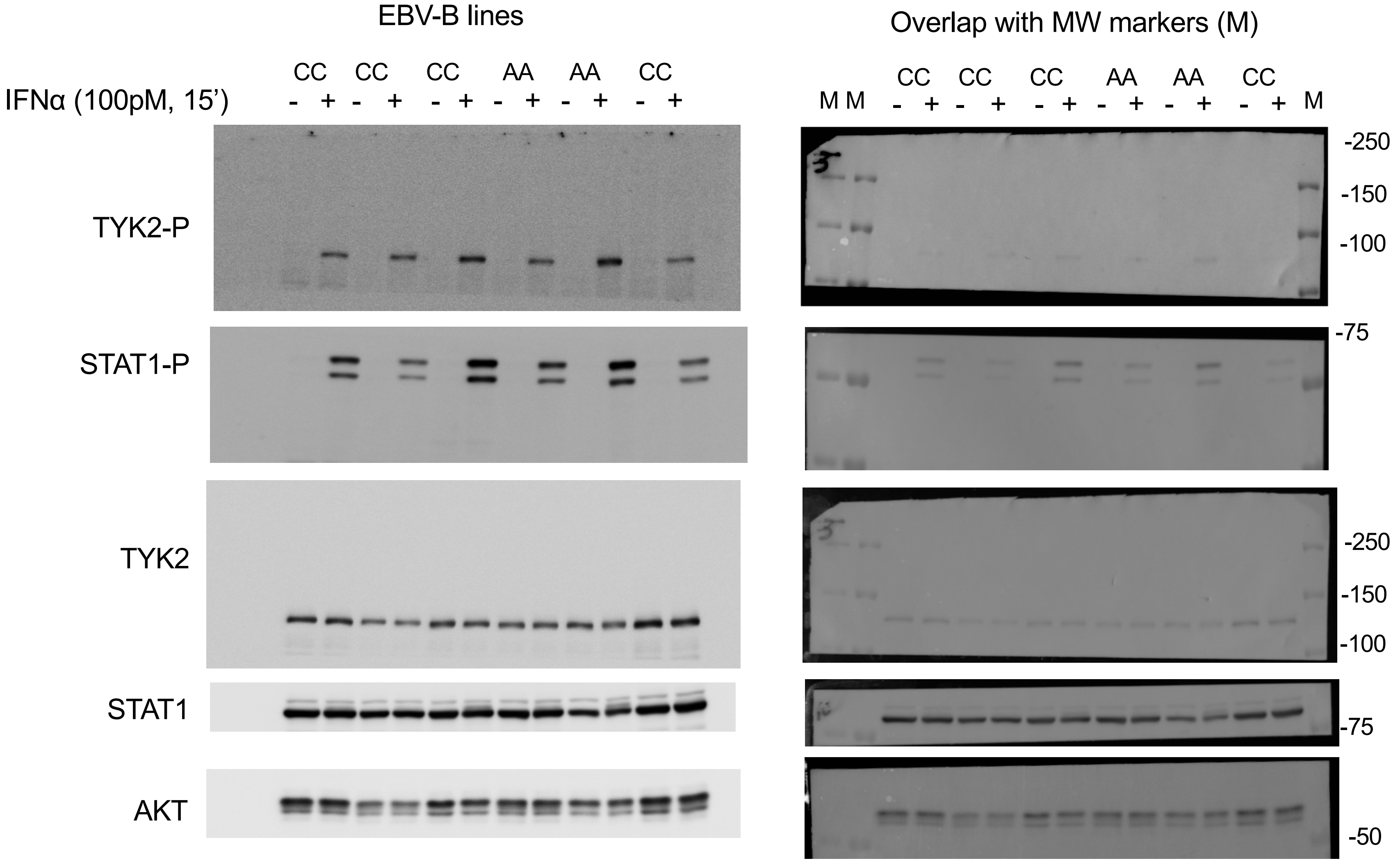

Fig. 2B (raw images)

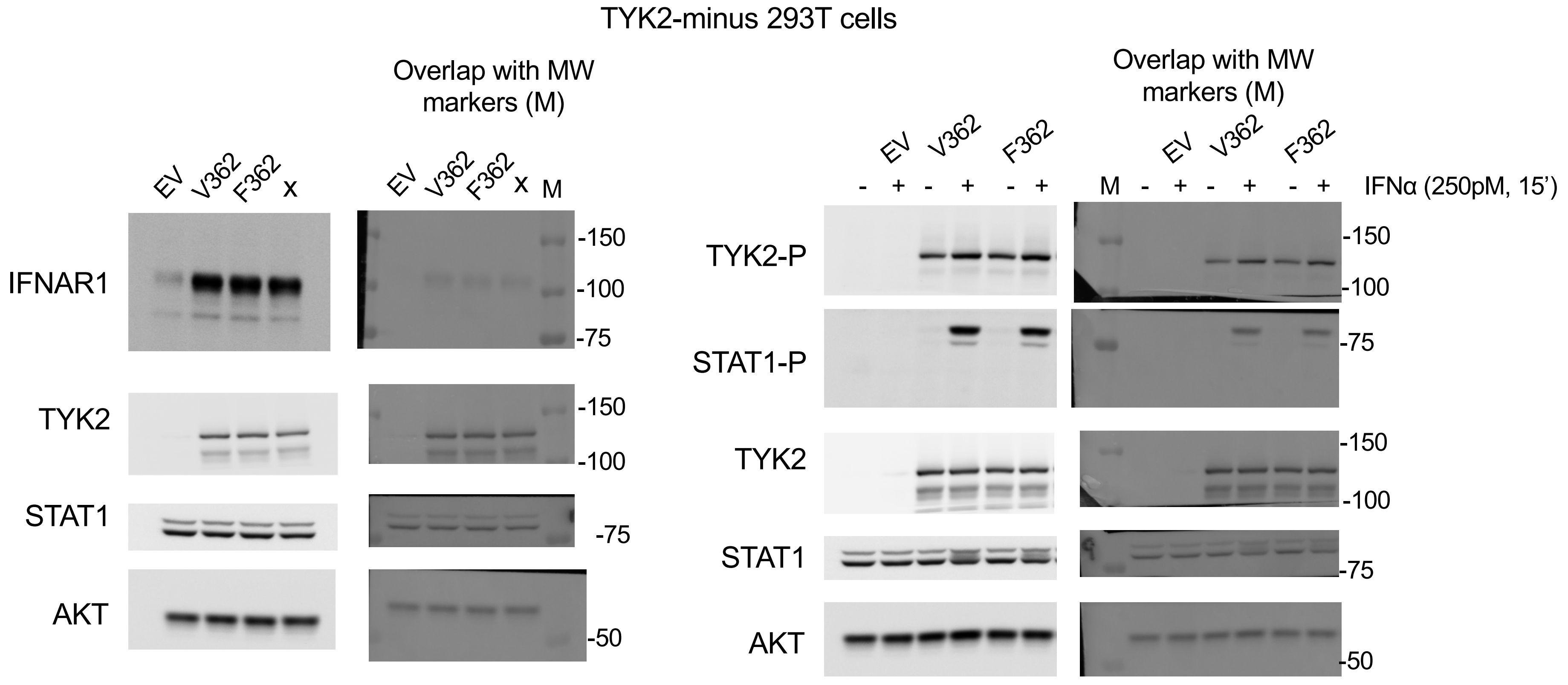

Fig. 2C (raw images)

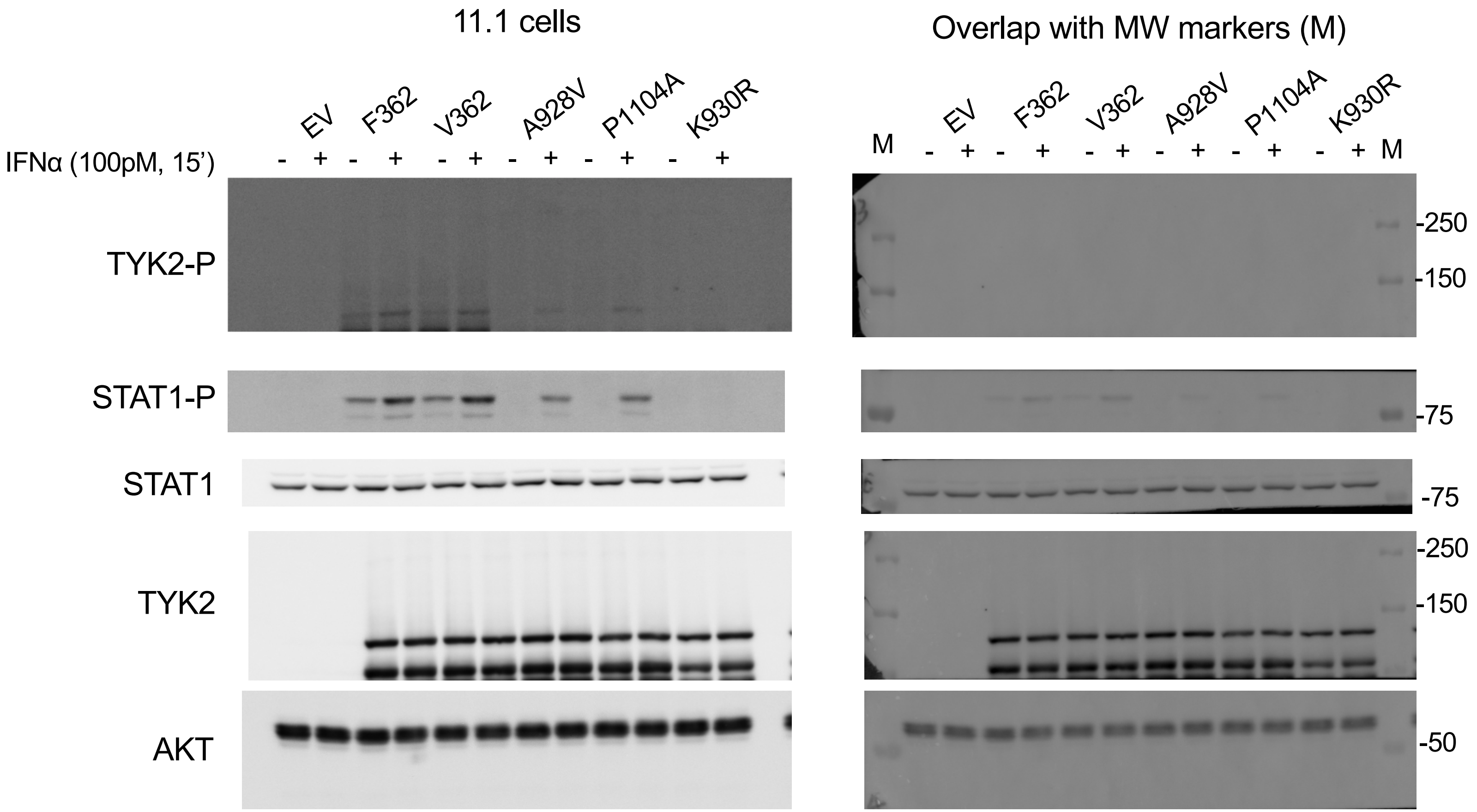

Fig. 4A (raw images)

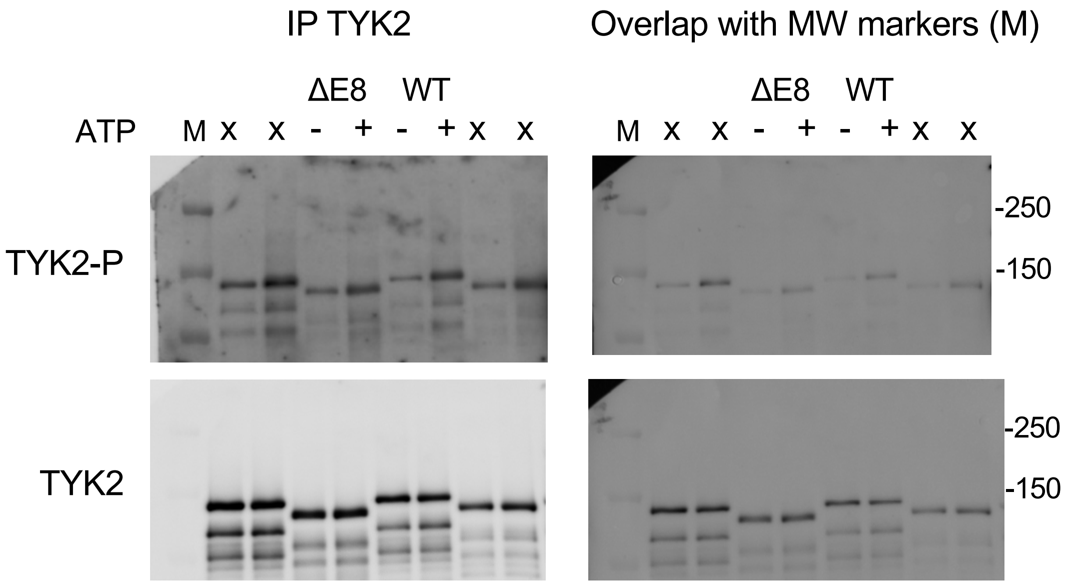

Fig. 4B (raw images)

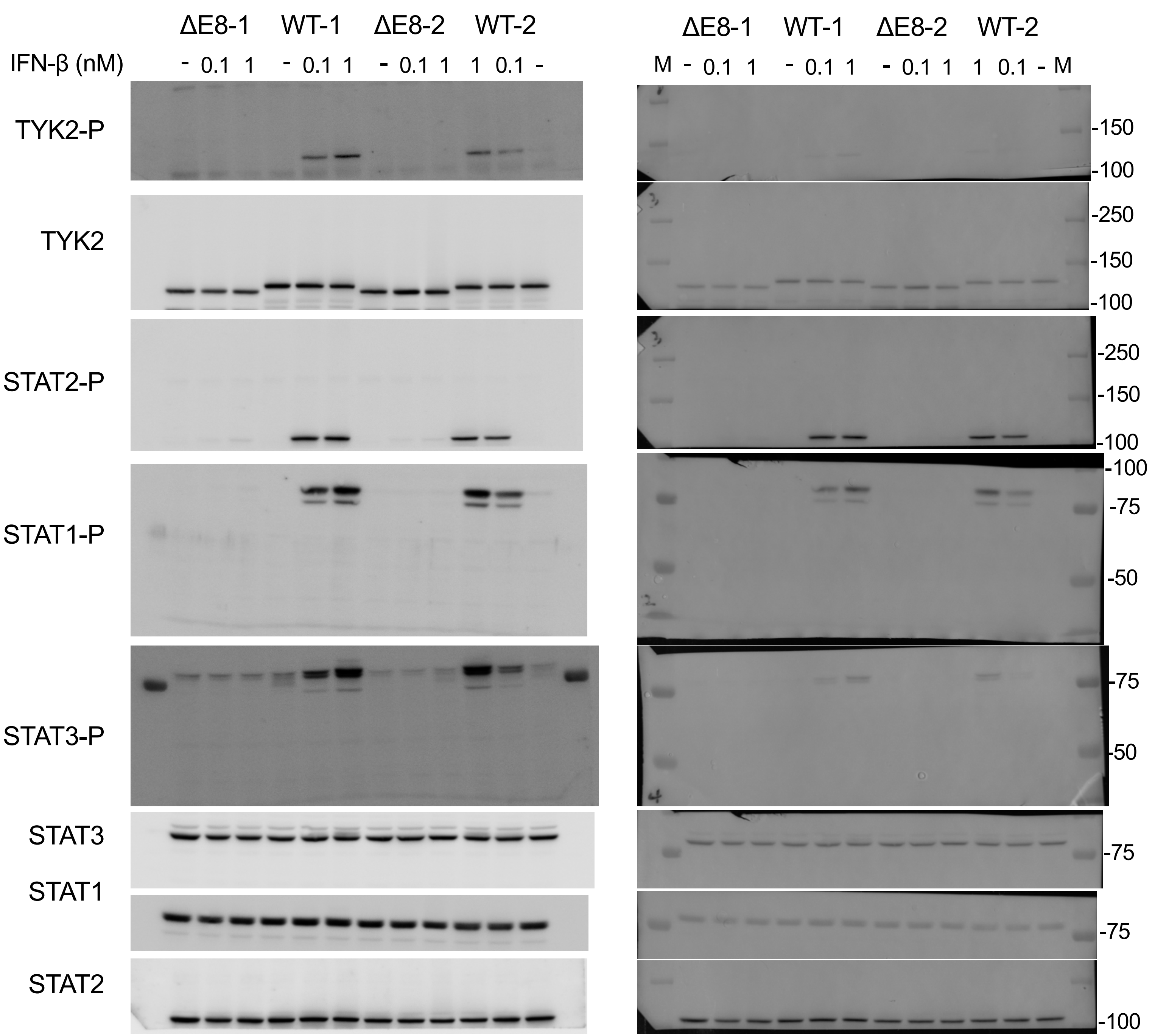

Fig. 4C (raw images)

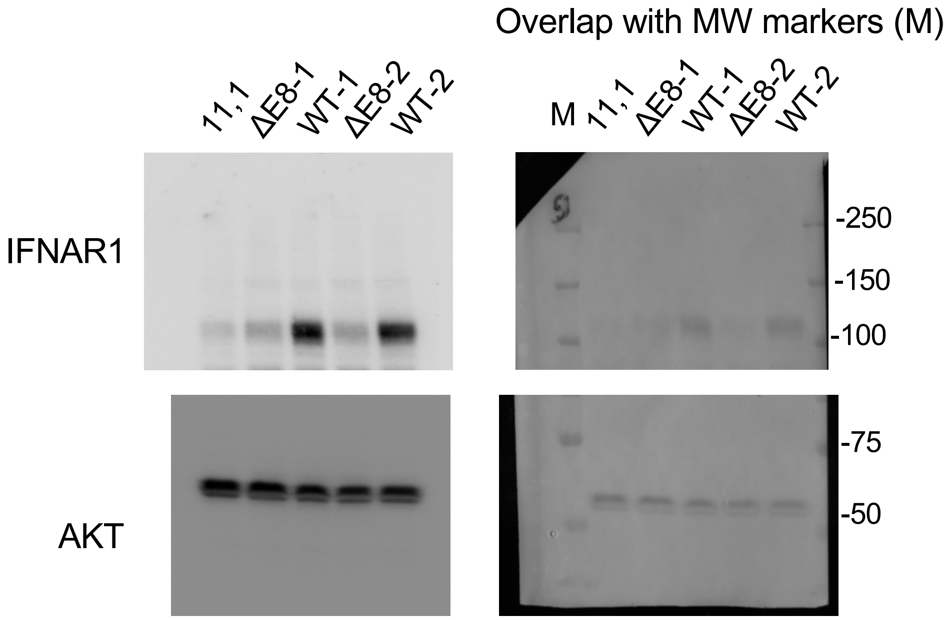

Fig. 4D (raw images)

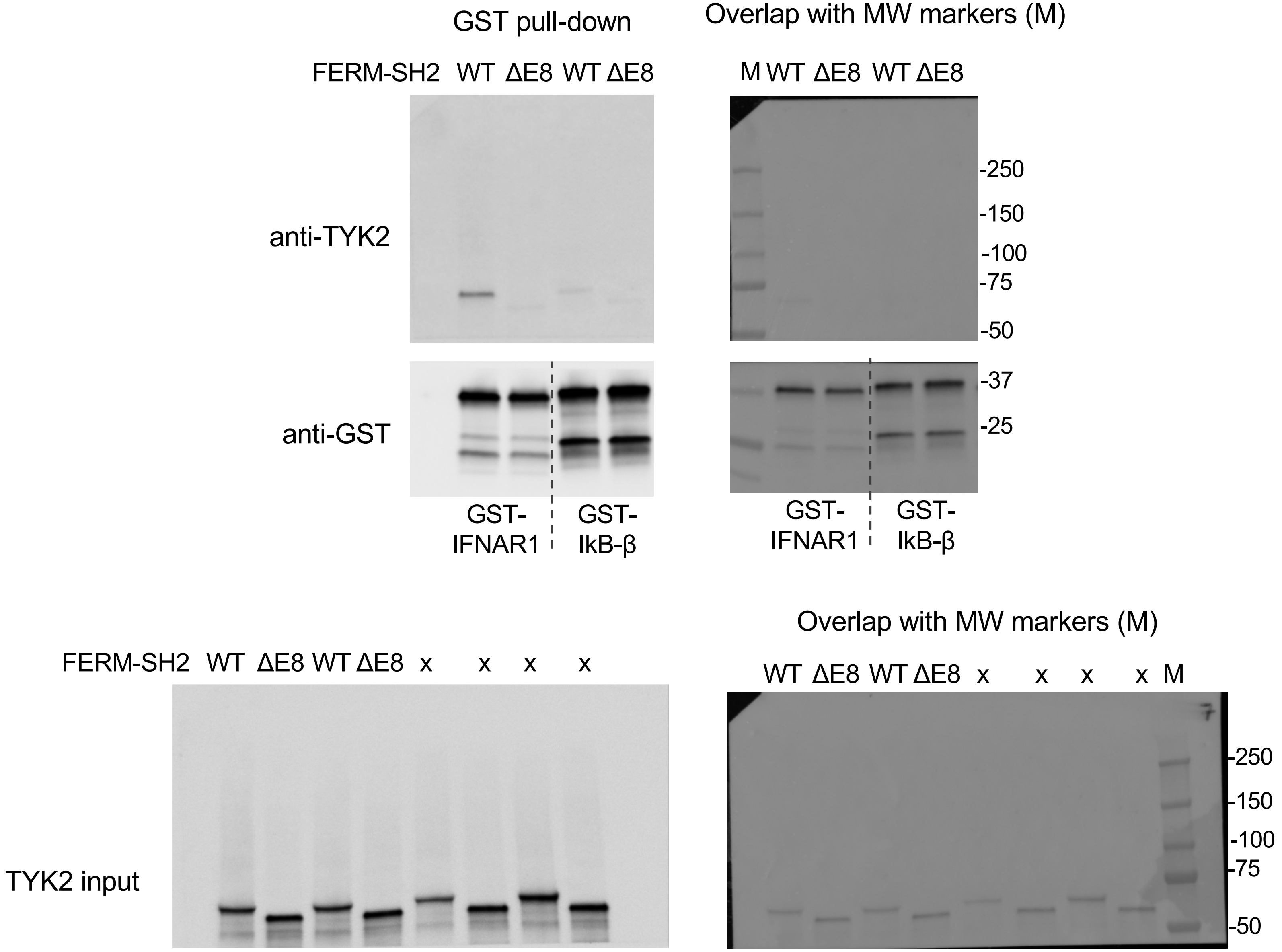

Fig. 4E (raw images)

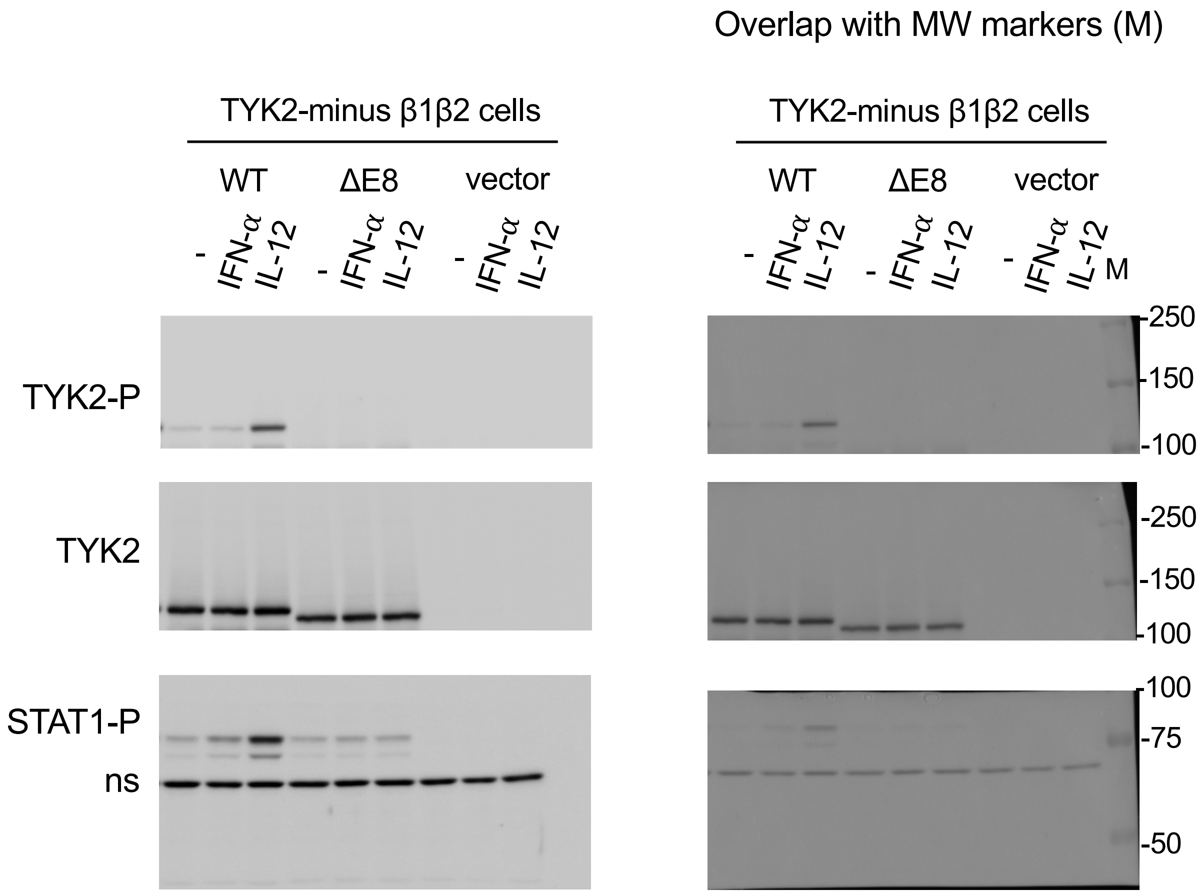

Fig. 4F (raw images)

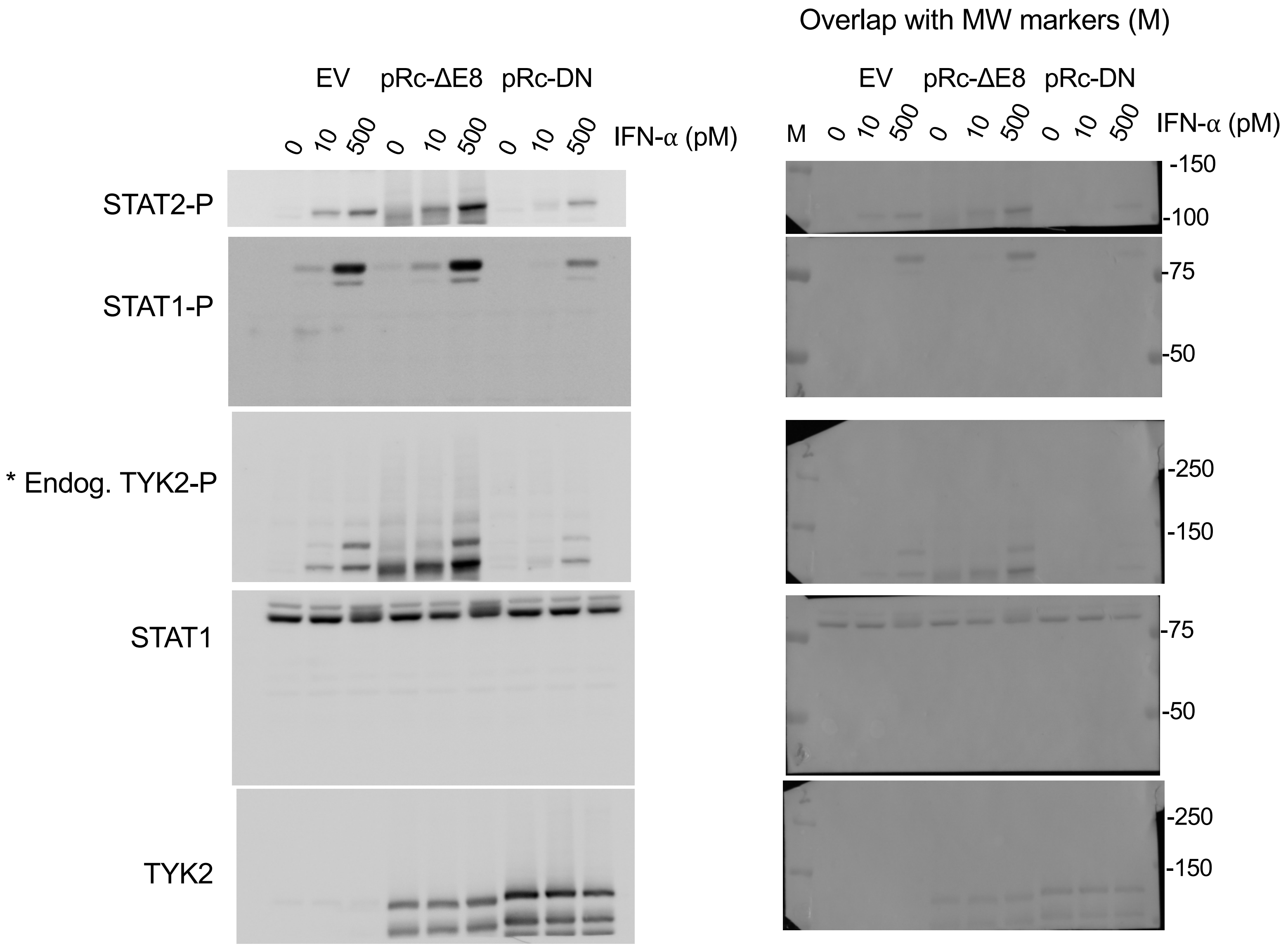

** anti-TYK2-P after mild stripping of STAT2-P*
