# Supplementary material for: Two common disease-associated TYK2 variants impact exon splicing and TYK2 dosage
Source: PLoS One. 2020 Jan 21;15(1):e0225289. doi: 10.1371/journal.pone.0225289 (PMC6974145; doi:10.1371/journal.pone.0225289)
Supplement: S2 Fig — Taken and adapted from Supplem. S3 Fig. in (Wallweber HJ et al, Nat. Struct. Mol. Biol. 2014. 21:443). Shown is the secondary structure of the TYK2 FERM-SH2 domain in complex with a short peptide of the IFNAR1 cytoplasmic portion (yellow). SH2 domain in light blue. The FERM comprises three lobes (F1, F2 and F3). Helices displayed as cylinders, strands displayed as block arrows, and loops displayed as lines. Arrows point to the start (5’) and the end (3’) of the exon 8-encoded segment, spanning from the β3-β4 loop to the β7 strand of the F3 lobe. Valine 362 is located in the unstructured β3-β4 loop and is tentatively indicated by an arrow. (PDF) [file pone.0225289.s003.pdf]

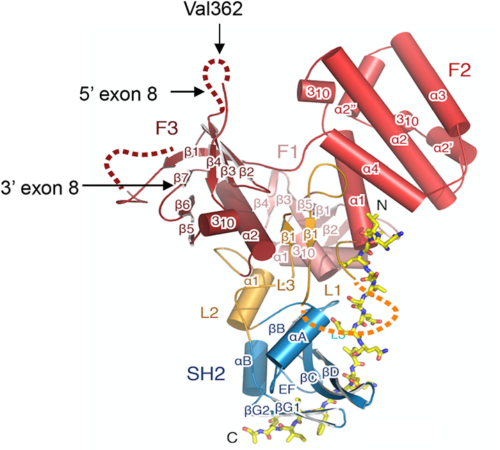

Adapted from Wallweber HJ *et al*, 2014

S2 Fig
